# Genetic polymorphisms in pre-miRNAs predict the survival of non-small-cell lung cancer in Chinese population: a cohort study and a meta-analysis

**DOI:** 10.18632/oncotarget.20276

**Published:** 2017-08-16

**Authors:** Lingzi Xia, Zhihua Yin, Xuelian Li, Yangwu Ren, Haibo Zhang, Yuxia Zhao, Baosen Zhou

**Affiliations:** ^1^ Department of Epidemiology, China Medical University, Shenyang, Liaoning, 110122, P.R. China; ^2^ Key Laboratory of Cancer Etiology and Prevention (China Medical University), Liaoning Province Department of Education, 110122, P.R. China; ^3^ Department of Radiotherapy, Shenyang North Hospital, Shenyang, Liaoning, 110001, P.R. China; ^4^ Department of Radiotherapy Oncology, The Fourth Affiliated Hospital of China Medical University, Shenyang, Liaoning, 110001, P.R. China

**Keywords:** non-small cell lung cancer, prognosis, microRNA, single nucleotide polymorphism, meta-analysis

## Abstract

**Background:**

To explore the association of genetic polymorphisms in pre-miRNA 30c-1 rs928508 and pre-miRNA 27a rs895819 with non-small-cell lung cancer prognosis.

**Materials and Methods:**

480 patients from five hospitals were enrolled in this prospective cohort study. They were followed up for five years. The association between genotypes and overall survival was assessed by Cox proportional hazards regression models. A meta-analysis was conducted to provide evidence for the effect of microRNA 27a rs895819 on cancer survival.

**Results:**

G-allele containing genotypes of microRNA 30c-1 polymorphisms and C-allele containing genotypes of microRNA 27a were significantly associated with poorer overall survival. Multivariate Cox regression models indicated that these genetic polymorhpisms were independently predictive factors of poorer overall survival. In stratified analysis, the effect was observed in many strata. The significant joint effect was also observed in our study. Patients with G allele of microRNA 30c-1 rs928508 and C allele of microRNA 27a rs895819 had the poorer overall survival than patients with C allele of rs928508 and T allele of rs895819. The effect of the microRNA 27a rs895819 on non-small cell lung cancer overall survival was supported by the meta-analysis results.

**Conclusions:**

The two single nucleotide polymorphisms in microRNA 30c-1 and microRNA 27a can predict the outcome of non-small cell lung cancer patients and they may decrease the sensitivity to anti-cancer drugs.

## INTRODUCTION

Lung cancer is a malignancy worldwide with complicated, multi-factorial aetiology, involving both environmental and genetic factors. Tobaccon exposure is a widely recognized risk factor for development of lung cancer. Morbidity and mortality of lung cancer increase in a constant rate in both genders [1] in China. And lung cancer is the most common cause of cancer death in China [2, 3]. With an increasing number of inpatients and a much low 5-year-survival, lung cancer imposes a heavy burden on both family and society in China [4–6]. Much effort was taken to identify prognostic biomarkers. A recent focus arised in the field of epigenetics, which also includes silencing of target genes with microRNAs (miRNAs).

MiRNAs are a class of 19–25 nt in length, small non-coding RNAs, which normally disturb the stability or translation of the target gene by pairing with the mRNAs [7]. For one single miRNA, it may target to tens of mRNAs. The biological functions of most miRNAs may be marvelous. Genetic variants presented in miRNA genes and processing mechanisms may alter miRNA expression and maturation [8]. Moreover, single nucleotide polymorphisms (SNPs) in miRNAs and its binding site may influence the affinity between miRNAs and mRNAs [9]. Thus genetic polymorphisms in miRNAs may influence the cancer prognosis either by affecting the maturation or by altering the ability to combine with target mRNAs.

Two miRNAs, microRNA 27a and microRNA 30c-1, were observed in many pivotal cancer progressions, such as invasion [10–12] and response to chemotherapy [13–16]. Accumulating evidence has shown that aberrant expression profiles and genetic polymorphisms of microRNA 27a [17–21] and microRNA 30c-1 [22, 23] are associated with cancer survival. rs928508 in pre-miRNA 30c-1 was the SNP site identified in 2010 in Hu's study [22]. And the effect of this polymorphism on lung cancer survival needs to be explored. The role of rs895819 in pre-miRNA 27a in various cancers had been discussed in some studies. But the role in lung cancer survival received little exploration [18, 21]. In this present study, we investigated the effect of the two genetic polymorphisms in pre-miRNA 30c-1 and pre-miRNA 27a on the prognosis of non-small cell lung cancer (NSCLC).

## RESULTS

There are no differences between follow-up group and lost to follow-up group (data were shown in Supplementary Table 1). The baseline characteristics of lung cancer patients are listed in Table 1. All of the patients are females. We set 60 as the age category boundary. Patients less than 60 are set as one category and the others are set as another category. More than half of the patients are non-smokers and most of the subjects are NSCLC. No small cell lung cancer is included in our present study. About 67% of the female patients are diagnosed with cancer in a relatively later clinical stage. The majority of patients received chemotherapy or surgery after they are diagnosed with lung cancer.

**Table 1:** The baseline characteristics of patients

Characteristics	No. of patients (%)	Death	%	*P*-value	MST*	Log-rank *P*-value
Age						
< 60	248 (54.6)	213	85.9	0.991	25.72	0.293
≥ 60	206 (45.4)	177	85.9		23.65	
Tabaccon exposure						
No	252 (55.5)	212	84.1%	0.224	25.31	0.064
Yes	202 (44.5)	178	88.1%		22.85	
Histological type						
AD	237 (52.2)	196	82.7	0.101	22.20	< 0.001
SQU	164 (36.1)	148	90.2		23.54	
Others	53 (11.7)	46	86.8		36.59	
Clinical stage						
I	95 (20.9)	65	68.4	< 0.001	43.82	< 0.001
II	56 (12.2)	48	85.7		23.65	
III	263 (57.9)	240	91.3		17.38	
IV	40 (8.8)	37	92.5		19.96	
Chemotherapy						
No	22 (4.8)	20	90.9	0.754^#^	14.82	0.006
Yes	432 (95.2)	370	85.6		25.09	
Surgery						
No	123 (27.1)	112	91.1	0.054	20.59	0.006
Yes	331 (72.9)	278	84.0		25.42	

*MST:median survival time, AD:adenocarcinoma, SQU: squamous carcinoma.

^#^Fisher exact *P* value.

**Figure 1:**
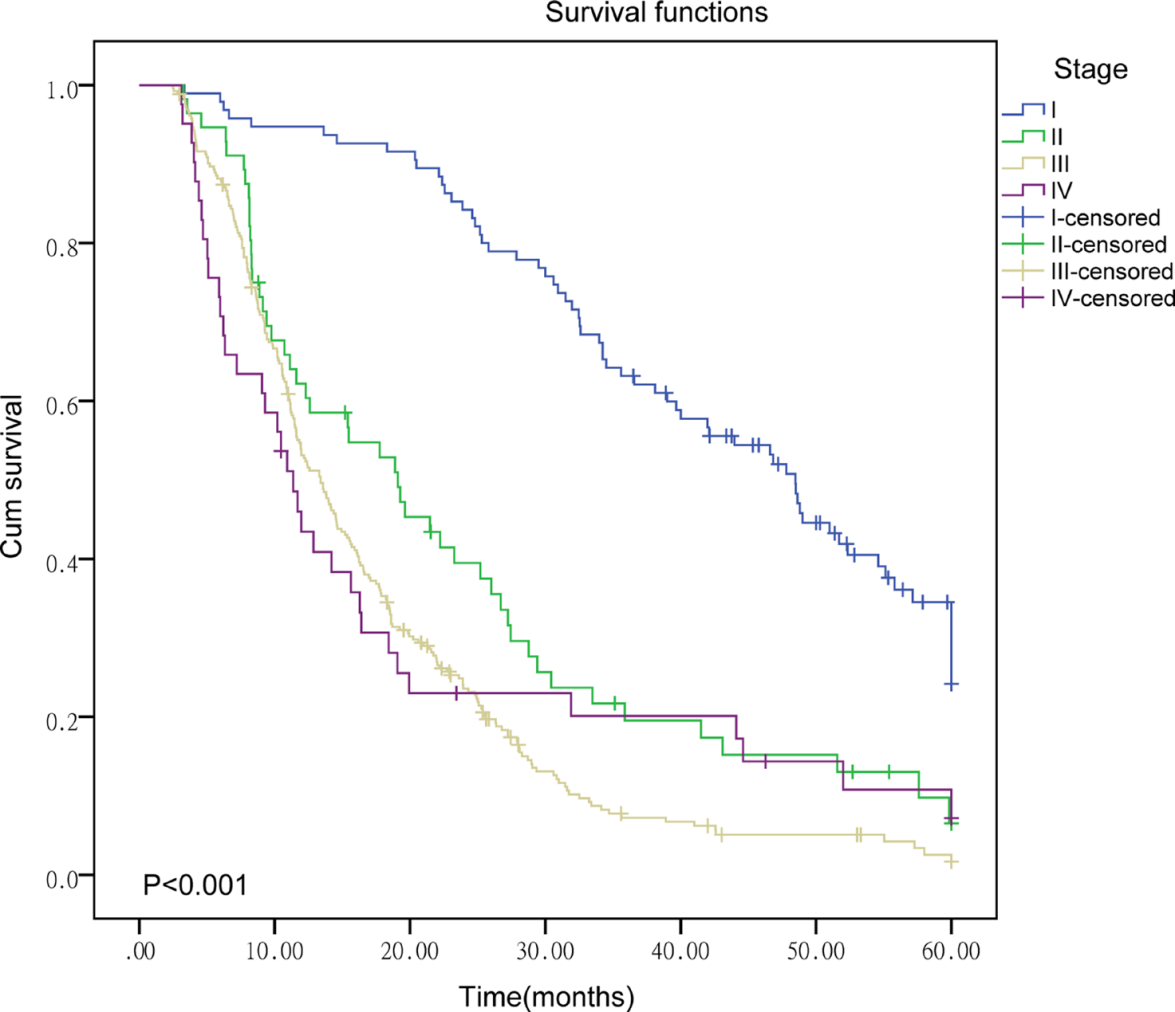
Survival curve of patients in different clinical stages

As shown in Table 1, the mortality and median survival time (MST) between patients with different clinical stages are statistically significant (*P* < 0.001). For late-stage patients, the mortality is higher and the MST is shorter than early-stage patients. Survival curves for patients with different clinical stages are presented in Figure 1. Significant differences in MST between patients with different histological types and therapeutic strategies are also observed (*P* < 0.006). Patients receiving chemotherapy or surgery can live longer (*P* = 0.006).

**Figure 2:**
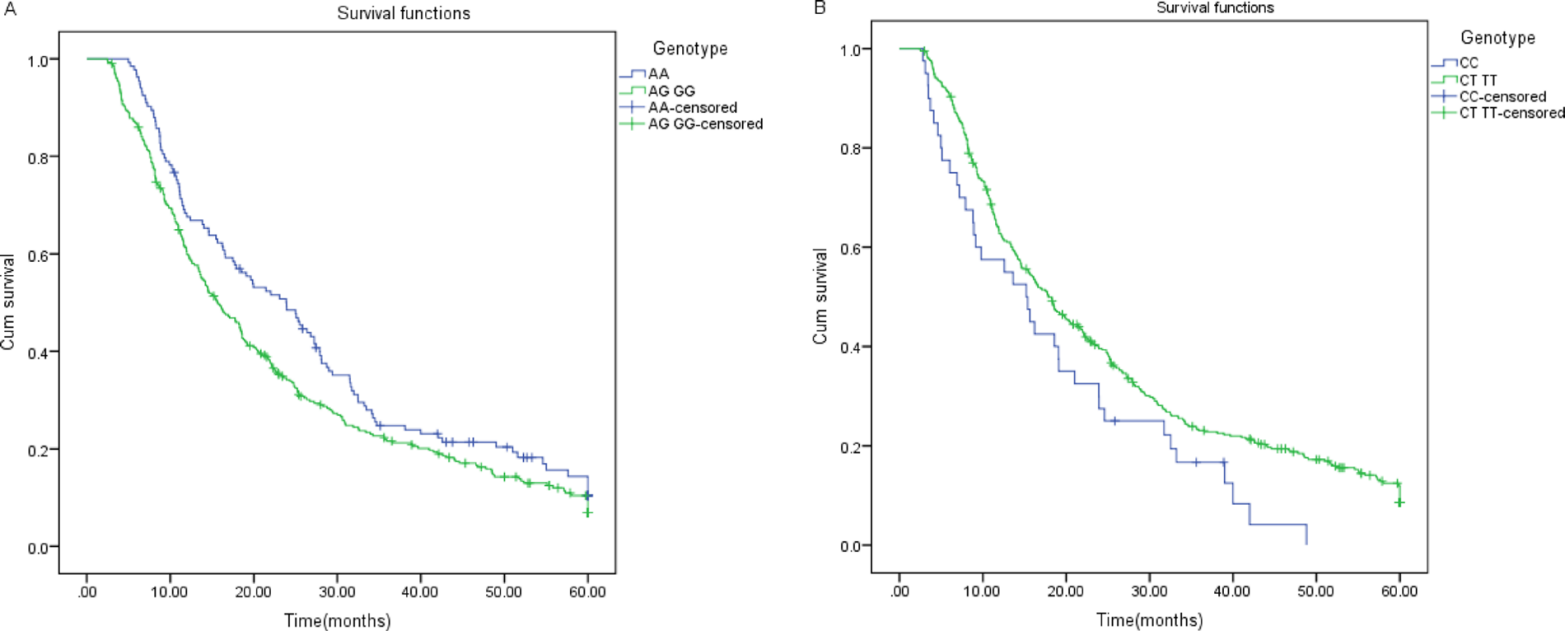
Survival curve of patients with different genotypes (**A**) The survival curve of patients with different genotypes of microRNA 30c-1 (*P* = 0.023). (**B**) The survival curve of patients with different genotypes of microRNA 27a (*P* = 0.029).

Results for the effect of the two SNPs on cancer overall survival (OS) are listed in Table 2. We observe that G-allele containing genotypes of microRNA 30c-1 rs928508 polymorphisms and C-allele containing genotypes of microRNA 27a rs895819 polymorphisms were significantly associated with poorer survival. Results of multivariate Cox proportional hazards regression models imply that both SNPs were independent predictive factors of poor prognosis. The survival curves are described in Figure 2.

**Table 2:** The association of polymorphisms in microRNA 30c-1 and microRNA 27a with overall survival

SNP	Genotypes	Num	MST	Log-rank *P* value	HR	95% CI (*P* value)	aHR	95% CI (*P* value)
microRNA 30c-1	AA	133	28.44	0.054	1.00		1.00	
rs928508	AG	229	23.20		1.28	1.01–1.61 (0.041)	1.30	1.03–1.65 (0.027)
	GG	92	21.95		1.37	1.03–1.83 (0.033)	1.45	1.08–1.94 (0.012)
	AG	229	23.20	0.592	1.00		1.00	
	GG	92	21.95		1.07	0.83–1.39 (0.593)	1.09	0.84–1.43 (0.514)
	AA/AG	362	25.25	0.188	1.00		1.00	
	GG	92	21.95		1.18	0.92–1.51 (0.190)	1.24	0.96–1.59 (0.097)
	AA	133	28.44	0.019	1.00		1.00	
	AG/GG	321	22.86		1.30	1.04–1.62 (0.020)	1.34	1.07–1.67 (0.010)
	A allele	495	26.04	0.022	1.00		1.00	
	G allele	413	22.63		1.18	1.02–1.36 (0.023)	1.21	1.05–1.39 (0.009)
microRNA 27a	TT	247	26.27	0.033	1.00		1.00	
rs895819	CT	167	23.71		1.17	0.95–1.45 (0.143)	1.23	0.99–1.52 (0.062)
	CC	40	18.25		1.56	1.09–2.22 (0.014)	1.71	1.20–2.45 (0.003)
	CT	167	23.71	0.100	1.00		1.00	
	CC	40	18.25		1.35	0.94–1.95 (0.102)	1.44	1.00–2.08 (0.050)
	CT/TT	414	25.23	0.030	1.00		1.00	
	CC	40	18.25		1.46	1.04–2.05 (0.031)	1.57	1.11–2.21 (0.010)
	TT	247	26.27	0.041	1.00		1.00	
	CC/CT	207	22.79		1.23	1.01–1.50 (0.042)	1.30	1.06–1.50 (0.012)
	T allele	661	25.62	0.011	1.00		1.00	
	C allele	247	22.14		1.22	1.05–1.43 (0.012)	1.28	1.09–1.49 (0.002)

*Num: the patients number for each genotype, aHR: adjusted HR for histological type, clinical stage and, receipt of chemotherapy and surgery.

Results of the stratified analysis are summarized in Supplementary Table 2. The significant effect for microRNA 30c-1 rs928508 and microRNA 27a rs895819 polymorphisms can be observed in many strata. Both the two SNPs can be prognostic in smokers, lung adenocarcinoma patients, squamous cell carcinoma patients, late stage patients and patients receiving chemotherapy.

Results in stratified analysis imply the joint effect of the two SNPs on prognosis. We proceed to analyse the joint effect. Results are shown in Table 3. As shown in Table 3, we observe the joint effect of the two SNPs on cancer prognosis in many strata. The more risk alleles, the poorer OS (larger HRs with smaller *P* values).

**Table 3:** The joint effect of the two SNPs

Stratum	Model*	Cases	HR	95% CI	*P* value
Smokers	0	39	1.00		
	1	71	1.29	0.83–2.00	0.252
	2	67	1.66	1.07–2.59	0.024
	3 + 4	25	3.00	1.75–5.13	< 0.001
AD	0	48	1.00		
	1	85	1.24	0.93–1.86	0.289
	2	68	1.30	0.85–1.98	0.226
	3 + 4	36	2.20	1.35–3.58	0.001
SQU	0	26	1.00		
	1	58	0.84	0.51–1.40	0.505
	2	58	1.64	0.99–2.72	0.054
	3 + 4	22	1.85	1.01–3.39	0.047
III/IV	0	47	1.00		
	1	108	1.22	0.85–1.75	0.290
	2	107	1.53	1.06–2.19	0.023
	3 + 4	41	2.00	1.30–3.09	0.002
Chemotherapy	0	80	1.00		
	1	154	1.15	0.85–1.55	0.364
	2	138	1.41	1.04–1.91	0.029
	3 + 4	60	1.67	1.16–2.41	0.006

*0:AA of microRNA 30c-1 polymorphisms and TT of microRNA 27a polymorphisms, 1: AG of microRNA 30c-1 polymorphisms and TT of microRNA 27a polymorphisms, or AA of microRNA 30c-1 polymorphisms and CT of microRNA 27a polymorphisms, 2: GG of microRNA 30c-1 polymorphisms and TT of microRNA 27a polymorphisms, or AA of microRNA 30c-1 polymorphisms and CC of microRNA 27a polymorphisms, or AG of microRNA 30c-1 polymorphisms and CT of microRNA 27a polymorphisms, 3: AG of microRNA 30c-1 polymorphisms and CC of microRNA 27a polymorphisms, or GG of microRNA 30c-1 polymorphisms and CT of microRNA 27a polymorphisms, 4:AA of microRNA 30c-1 polymorphisms and CC of microRNA 27a polymorphisms.

**Figure 3:**
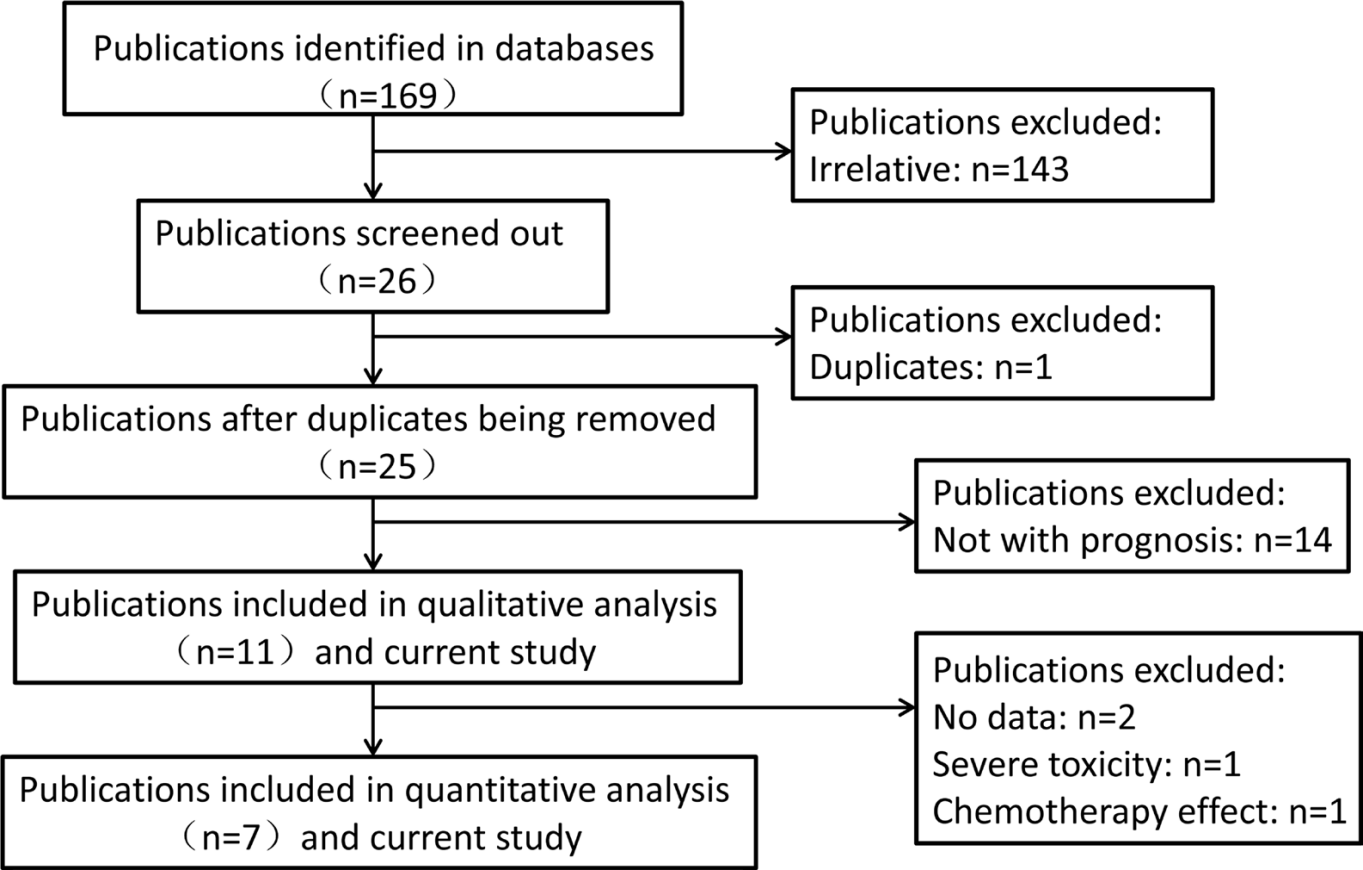
The workflow of the enrollment in the meta-analysis

With only two publications, we didn't conduct the meta-analysis for the relationship between cancer prognosis and microRNA 30c-1 rs928508. Publications included in this analysis for microRNA 27a rs895819 are exhibited in Table 4. The workflow of the enrollment is described in Figure 3. Finally, eleven [18, 21, 24–32] eligible publications were screened out. Among them, eight publications focus on the relationship with OS and two focus on recurrence-free survival (RFS). Tumor types of these publications include lung cancer, gastric cancer, breast cancer, B-cell lymphoma, gallbladder cancer and colorectal cancer. Seven [18, 21, 24, 27, 28, 30, 32] publications and our current study were included in quantitative analysis.

**Table 4:** The information of the publications enrolled in this meta-analysis

Year	Name	Tumor type	Population	Number	Outcome
2017[24]	Xu	GC	China	939	OS
2015[27]	Ma	NSCLC	China	560	RFS, OS
2013[18]	Xu	NSCLC	China	576	OS
2013[28]	Stenholm	GC	Germany	674	OS
2012[21]	Yoon	NSCLC	Korea	388	RFS
2013[30]	Zhang	BCL	China	100	OS
2015[32]	Gupta	GBC	Indian	606	OS
Current study	Xia	NSCLC	China	454	OS

GC:gastric cancer, NSCLC:non-small cell lung cancer, BCL: B-cell lymphoma, GBC:gallbladder cancer.

According to the publications’ results, C-allele containing genotypes of microRNA 27a rs895819 are related to gastrointestinal toxicity in more than 56 years of age, smoking and non-smoking lung cancer patients that received platinum-based chemotherapy [25]. No significant association with disease-free survival was observed in breast cancer [29]. No significant association with 5FU chemotherapy effect was observed in colorectal cancer patients [31]. The results for the association with cancer OS are inconsistent.

**Figure 4:**
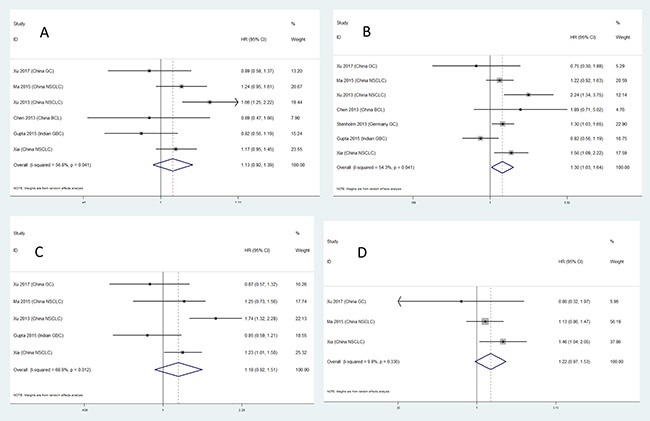
The forest plots for the relationship between overall survival and cancer prognosis (**A**) AG vs AA, (**B**) GG vs AA, (**C**) AG + GG vs AA, (**D**) GG vs AA + AG.

**Figure 5:**
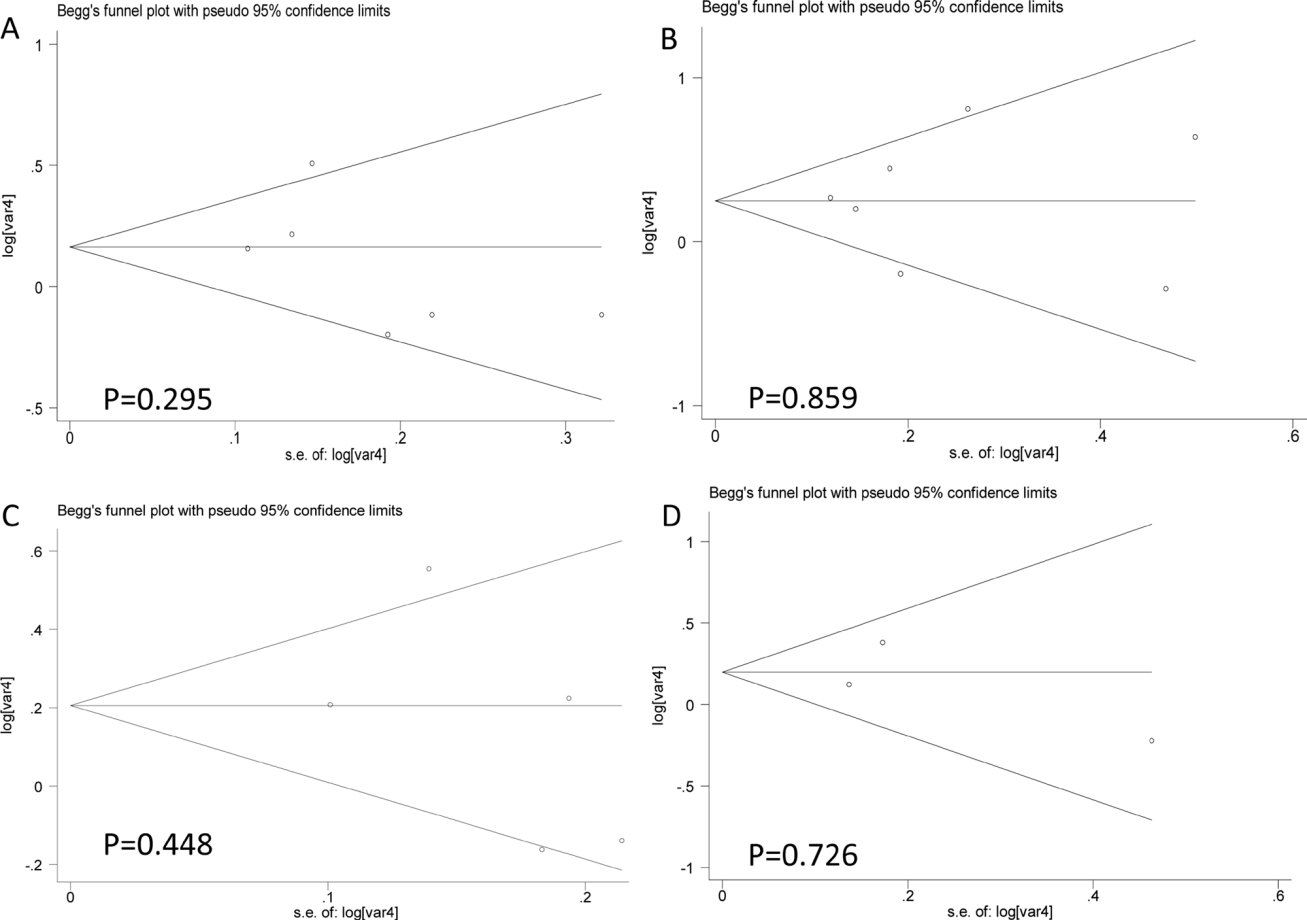
The funnel plots for the relationship between overall survival and cancer prognosis (**A**) AG vs AA, (**B**) GG vs AA, (**C**) AG + GG vs AA, (**D**) GG vs AA + AG.

**Figure 6:**
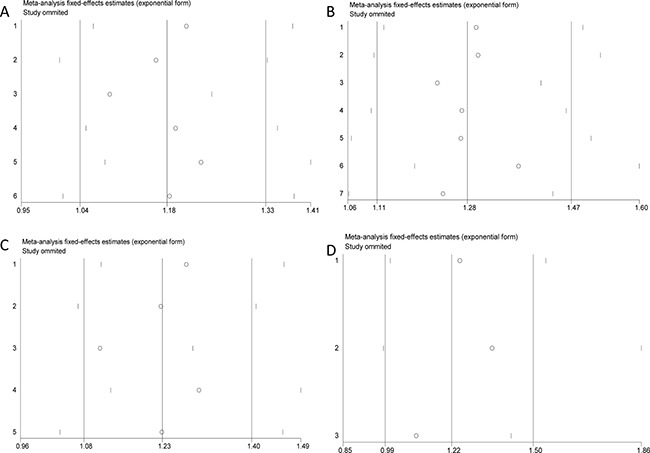
Sensitivity analysis results for the relationship between overall survival and cancer prognosis (**A**) AG vs AA, (**B**) GG vs AA, (**C**) AG + GG vs AA, (**D**) GG vs AA + AG.

Results for meta-analysis are shown in Table 5. CC genotype of microRNA 27a rs895819 is associated with OS. Patients with CC genotype of microRNA 27a rs895819 may have a poorer survival. The forest plots are exhibited in Figure 4. The funnel plots are exhibited in Figure 5. Sensitivity analysis results are exhibited in Figure 6. No significant association is observed in our meta-analysis for the relationship between RFS and rs895819. No publication bias exists in this study.

**Table 5:** Pooled HR and 95% CIs of microRNA 27a rs895819

Outcome	Model	No. of studies	No. of patients	HR (95% CI)	*P*-value	Heterogeneity (*I*^2^, *P*-value)
OS	CT vs TT	6	3235	1.133 (0.923, 1.391)	0.232	56.8%, 0.041
	CC vs TT	7	3909	1.296 (1.027, 1.636)	0.029	54.3%, 0.041
	CC + CT vs TT	5	3135	1.176 (0.916, 1.509)	0.204	68.9%, 0.012
	CC vs CT + TT	3	1953	1.220 (0.975, 1.526)	0.082	9.8%, 0.330
RFS	CT vs TT	2	948	1.117 (0.892, 1.400)	0.335	0.0%, 0.891
	CC vs TT	2	948	0.979 (0.762, 1.256)	0.866	0.0%, 0.558
	CC + CT vs TT	2	948	1.091 (0.899, 1.323)	0.380	0.0%, 0.791

OS: overall survival, RFS: recurrence-free survival.

**Figure 7:**
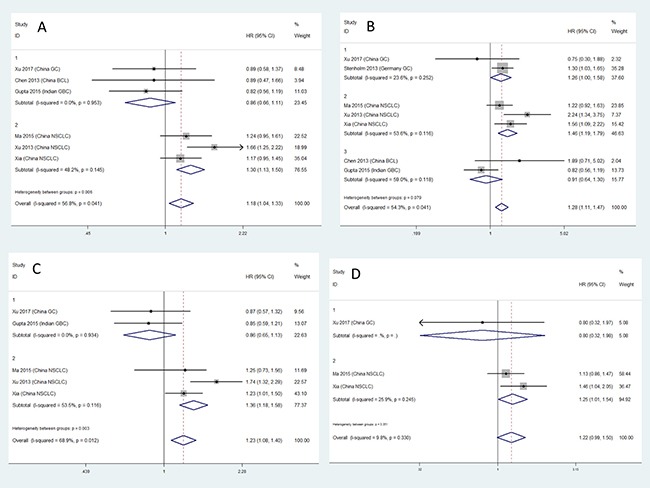
Forest plots for stratified analysis (**A**) AG vs AA, (**B**) GG vs AA, (**C**) AG + GG vs AA, (**D**) GG vs AA + AG.

Stratified analysis results are shown in Table 6. In this study, we conducted the stratified analysis according to tumor types. The C-allele containing genotypes of microRNA 27a rs895819 are associated with poorer NSCLC prognosis. CC genotype of microRNA 27a rs895819 is associated with poorer gastric cancer prognosis. The forest plots are exhibited in Figure 7.

**Table 6:** Stratified analysis of the relationship between microRNA 27a rs895818 and overall survival

Model	Subgroup	No. of studies	No. of patients	HR (95% CI)	*P-* value
CT vs TT	NSCLC	3	1590	1.298 (1.125, 1.498)	< 0.001
CC vs TT	NSCLC	3	1590	1.457 (1.187, 1.787)	< 0.001
	GC	2	1613	1.260 (1.004, 1.583)	0.046
CT + CC vs TT	NSCLC	3	1590	1.364 (1.177, 1.581)	< 0.001
CC vs CT + TT	NSCLC	2	1014	1.247 (1.010, 1.539)	0.040

NSCLC: non-small cell lung cancer, GC: gastric cancer.

## DISCUSSION

Once detected, the two miRNAs were observed to be involved in pivotal processes that may lead to poor cancer prognosis. Downregulation of microRNA 30c can promote the invasion of non-small cell lung cancer [10]. Downregulation of microRNA 30c was observed in drug-resistant cancer cells [13]. The polymorphisms in microRNA 30c-1 can alter the expression of mature microRNA 30c. The expression of pre-miR-30c and mature microRNA -30c is higher for AG/GG than AA [33]. All above indicated that the SNP of microRNA 30c-1 may influnce the cancer prognosis by altering the expression of mature microRNA 30c-1. In our study, we observed the poorer OS of patients with G-allele containing genotypes of microRNA 30c-1.

MicroRNA 27a could function to reverse multiple drug resistance by inhibiting FZD7/beta-catenin pathway in hepatocellular carcinoma and therefore promote the therapeutic effect in patients receiving chemotherapy in Chen's study [15]. The similar observation presented in Noratto's study [16] and the role of microRNA 27a was played through microRNA-27a-ZBTB10-Sp axis in colon cancer. The downregulation of microRNA 27a was essential for angiogenesis. And VE-cadherin was observed to be the dominant target of microRNA 27a *in vivo* and *in vitro* [34]. Rs10719 T to G substitution in Drosha 3′UTR was observed to result in the disruption of binding activity with microRNA 27a/b [35]. And A allele of microRNA 27a rs11671784 was related to decreased microRNA 27a expression [36]. All the results above indicated the effect of microRNA 27a SNP on therapeutic effect and survival. In our present study, C-allele containing genotypes of microRNA 27a were associated with poorer OS. Results are supported by the meta-analysis results.

In a previous study containing a relatively larger sample size, the protective effect of AG or GG of microRNA 30c-1 was observed on NSCLC survival, especially in older, early-stage and surgically resected patients [22]. In our study, the risk effect of AG or GG of microRNA 30c-1 was observed on NSCLC survival, especially in late-stage and squamous cell carcinoma patients. The effect of the SNP is not consistent in younger, smokers, adenocarcinoma and patients with chemotherapy. The variant allele-containing genotypes of microRNA 27a may function as risk factor for OS in gastric cancer patients in Germany as described in a previous study [28]. In Yoon's study [21], no significant effect of microRNA 27a polymorphisms on recurrence-free survival for NSCLC was observed. Xu [18] observed a risk effect of G-allele containing genotypes of microRNA 27a on OS in Chinese NSCLC patients, which is consistent with our results. Moreover, we observed the joint effect of the two SNPs on cancer prognosis in many strata.

There are some strengths and limitations in our study. One of the strengths is that this is a prospective cohort study indicating that our results are more reliable. Another one is that all of the patients are not included in a single hospital, which indicates the representativity of our study. The third one is that, to the best of our knowledge, this is the first meta-analysis concerning the association between microRNA 27a rs895819 and cancer prognosis. The forth one is that the meta-analysis is reliable and stable. One limitation of this study is that the sample size in each stratum is small. Another one is that the majority of included patients are late-stage. This may introduce biases in our study. The third one is that the number of publications included in this meta-analysis is small.

## MATERIALS AND METHODS

### Study population and follow-up

This study was a prospective cohort study and approved by the institutional review board of China Medical University and all subjects signed a written informed consent form. All subjects were females and they were from unrelated ethnic Han Chinese. Estimates of the exposure to environmental factors were reported in previous study [37]. 480 patients were recruited during March 2010 to March 2013 at five Liaoning hospitals. All patients were histologically confirmed when they were enrolled. All subjects were interviewed and venous blood sample was obtained from each subject. Detailed baseline information including age, gender, tobaccon exposure, clinical stage, histological type, receipt of chemotherapy and receipt of surgery has been collected.

Subjects received telephone follow-up every three months after being diagnosed with lung cancer. We adopted at least one of the following methods to confirm the date of death: 1) data from Shenyang Center for Disease Control and Prevention (CDC) registry system for cause of death; 2) inpatient and outpatient medical records; 3) Death Registry System of Shenyang Public Security Bureau. The patients were followed up to death or April 2015. Totally, we collected complete survival data of 454 patients. The others are lost to follow-up. To avoid information bias, we adopted two methods. One is that all interviewers received training before the study was conducted. Another one is that two individuals record the data independently and the third one check up the data for any contradiction. The MST is 24.7 months in the ongoing study.

### Genotyping

Genomic DNA was extracted from peripheral blood samples using the phenol-chloroform method. The TaqMan allelic discrimination method was used to genotype the two SNPs. The samples were read and analyzed from the ABI 7500 Fast Sequence Detection System (Applied Biosystems, USA). The average genotype call rates for the two SNPs was 99.5%. About 10% of the samples were randomly selected for confirmation by repeat genotyping, and the results were 100% concordant.

### Searching strategy

This meta-analysis was carried out in accordance with the guidelines of the meta-analysis of the Observational Studies in Epidemiology group (MOOSE) [38]. Databases including PubMed, SCIE, WanFang and CNKI were searched to identify the publications concerning the association with microRNA 30c-1 rs928508 and microRNA 27a rs895819. The strategies are as follows: all fields or subjects or full-text or MeSH or keywords contain “microRNA 30c-1 OR miRNA 30c-1 OR miR 30c-1 OR rs928508 OR microRNA 27a OR miRNA 27a OR miR 27a OR rs895819” OR “cancer OR carcinoma OR tumor OR neoplasm” OR “prognosis OR survival”. The reference lists were searched, as well. The last time for search is June, 2017.

Including criteria: 1) focus on the association with either rs928508 or rs895819 and cancer prognosis; 2) HR and 95%CI are available; 3) publications written in English or Chinese.

Excluding criteria: 1) duplicated publications or data; 2) meeting abstract with vague information.

### Data extraction

Data extraction and logging were conducted by two authors individually. The third author will take part in if the data are inconsistent. Information including publication year, first author name, tumor type, study population, sample size, outcome, HRs and 95% CIs was extracted.

### Statistical analysis

Goodness-of-fit chi-square test is used to compare the differences in the distribution of deaths between groups with different baseline characteristics. The OS is calculated from the date at diagnosis to the date of last follow-up or death. Median survival time and survival curves were estimated by Kaplan-Meier method and analyzed by the means of log-rank test. Univariate and multivariate Cox proportional hazards regression models were used to estimate the crude and adjusted HRs and 95% CIs. Stratified analysis was adopted to control the confounding bias. Q statistics were used to assess heterogeneity. *P* value less than 0.05 is considered heterogeneous and the random-effects model will be used to calculate the pooled HRs and 95% CIs. Otherwise, the fixed-effects model will be used. *I*^2^ statistic was used to measure the percentage of the variation that is due to heterogeneity rather than to chance. Stratified analysis was conducted according to the tumor types. Funnel plots and Begg's test were used to evaluate the publication bias. Sensitivity analysis was conducted to evaluate the stability of the study. Statistically significant *P*-value is less than 0.05. All of the statistical analyses were performed in SPSS 17.0 and all *P*-values are two-sided. In our study, we selected six genetic models for cohort study and four genetic models for meta-analysis. The Bonferroni *P* value for multiple-comparison is 0.008 for the estimation of the association between SNPs and cancer prognosis. Bonferroni *P* value for joint effect estimation is 0.01. Bonferroni *P* value for meta-analysis is 0.006.

## CONCLUSIONS

The two SNPs of microRNA 30c-1 and microRNA 27a may decrease the sensitivity to anti-cancer drugs and are predictive of non-small cell lung cancer patients survival. The observations in our study imply the role of microRNA 30c-1 and microRNA 27a polymorphisms in NSCLC patients prognosis.

## SUPPLEMENTARY MATERIALS FIGURES AND TABLES





## Abbreviations

SNP: single nucleotide polymorphism
NSCLC: non-small cell lung cancer
HR: hazard ratio
CI: confidential interval
MST: median survival time
OS: overall survival
RFS: recurrence-free survival
FZD7: frizzled class receptor 7
ZBTB10: zinc finger and BTB domain containing 10
CDC: center for disease control and prevention
AD: adenocarcinoma
SQU: squamous carcinoma
GC: gastric cancer
BCL: B-cell lymphoma
GBC: gallbladder cancer
