# Supplementary material for: Genetic polymorphisms in pre-miRNAs predict the survival of non-small-cell lung cancer in Chinese population: a cohort study and a meta-analysis
Source: Oncotarget. 2017 Aug 16;8(44):77963–74. doi: 10.18632/oncotarget.20276 (PMC5652828; doi:10.18632/oncotarget.20276)
Supplement: Supplementary file 1 [file oncotarget-08-77963-s001.pdf]

Genetic polymorphisms in pre-miRNAs predict the survival of non-small-cell lung cancer in Chinese population: a cohort study and a meta-analysis

SUPPLEMENTARY MATERIALS

Supplementary Table 1: The basic characteristics between lost to follow-up group and follow-up group

variables	Lost to follow-up group	Follow-up group	*p*
Age ($\overline{X}$ ± SD)	58.31 ± 10.84	55.96 ± 11.64	0.474
Histological type			
AD	15	237	0.844
SQU	8	164	
others	3	53	
Clinical Stage			
I	7	95	0.710
II	4	56	
III	14	263	
IV	1	40	

Supplementary Table 2: The results for stratified analysis. See Supplementary_Table_2
